# A New Strategy for Patient-Specific Implant-Borne Dental Rehabilitation in Patients With Extended Maxillary Defects

**DOI:** 10.3389/fonc.2021.718872

**Published:** 2021-12-10

**Authors:** Philippe Korn, Nils-Claudius Gellrich, Philipp Jehn, Simon Spalthoff, Björn Rahlf

**Affiliations:** Department of Oral and Maxillofacial Surgery, Hannover Medical School, Hannover, Germany

**Keywords:** maxillary defect, patient-specific implants, postablative surgery, dental rehabilitation, dental implants

## Abstract

**Purpose of the Study:**

Patients undergoing ablative tumor surgery of the midface are faced with functional and esthetic issues. Various reconstructive strategies, such as implant-borne obturator prostheses or microvascular tissue transfer, are currently available for dental rehabilitation. The present study shows the first follow-up of patients treated with patient-specific implants (IPS Implants^®^ Preprosthetic) for the rehabilitation of extended maxillary defects following ablative surgery.

**Patients and Methods:**

All patients treated with patient specific implants due to postablative maxillary defects were included. 20 implants were placed in the 19 patients (bilateral implants were placed in one of the cases). In 65.75% of the cases, resection was performed due to squamous cell carcinoma. In addition to the primary stability, the clinical implant stability, soft tissue management, successful prosthodontic restoration, and complications were evaluated at a mean follow-up period of 26 months.

**Results:**

All patient-specific implants showed primary stability and were clinically stable throughout the observation period. Definitive prosthodontic restorations were performed in all patients. No implant loosening was observed. Major complications occurred only in previously irradiated patients with insufficient soft tissue conditions (*p* = 0.058). Minor complications such as exposure of the underlying framework or mucositis were observed, but they never led to failure of restorations or implant loss.

**Conclusions:**

Treatment of postablative maxillary defects with patient-specific implants offers a safe alternative with predictable results for full and rapid dental rehabilitation, avoiding time-consuming augmentation procedures and additional donor-site morbidity.

## Introduction

Ablative tumor surgery of the midface often leads to esthetic and functional limitations that burden patients physically as well as psychologically (1). In addition, reconstruction of these defects is challenging, and a wide variety of reconstructive procedures have been described (2–4). Management of extended maxillary defects symbolizes technical achievements in maxillofacial surgery over the last 100 years. It started with obturating intra-oral defects and the use of prosthesis, followed by techniques of pedicled and free tissue transfer (5, 6). However, the overall goal is the full dental rehabilitation of compromised patients with implant-borne prostheses. The technique of subperiosteally placed but not bone-anchored implants was described as early as the 1940s, but was subsequently abandoned due to high complication rates (7, 8). The most technically advanced concept to achieve this goal was introduced by Dennis Rohner and Beat Hammer by combining the idea of immediate dental rehabilitation following a prosthodontic backwards planning protocol with the insertion of conventional dental implants into the fibula; prelaminating the perimplant soft tissues around the fibula with skin grafts and in a second stage, harvesting of the fibula bone flap using patient-specific cutting guides to accomplish the backwards-planned design; and mounting the individual prostheses onto the osseointegrated dental implants with plate fixation of the microvascular bone flap and into the maxillary defect site (9, 10). This technique was first published in 2000 and has only two potential drawbacks: first, microvascular bone transfer is mandatory, and second, to achieve a stable result of the backwards planned dental rehabilitation concept, the patients’ biology has to comply without forming pseudarthroses in the area of the fibular “wedge” cuts as well as the contact zones between the bony recipient site and bone flap. However, in cases where the patient is not eligible for harvesting a microvascular bone flap, the whole technique cannot be used. Nevertheless, this concept was unique in times of analogous planning and was benchmarked as the most advanced concept for rehabilitating patients with extended maxillary or mandibular defects. Today, this protocol can be realized using virtual planning and digital 3D printing technology to fabricate patient-specific cutting guides or plates (11–13). In cases where bony reconstruction was either not possible or failed and conventional implants were not an option, the bail-out strategy limited to the lateral maxilla was the insertion of zygomatic implants to enable prosthodontic restoration (14).

Recently, subperiostally placed and multi-vector anchored patient-specific implants with immediate loading for a one-step reconstruction of the maxilla (IPS Implants^®^ Preprosthetic, KLS Martin, Tuttlingen, Germany) have become available (15, 16). The authors present the first follow-up of patients treated with this type of implants following ablative surgery of the maxilla.

## Patients and Methods

**Figure 1:**
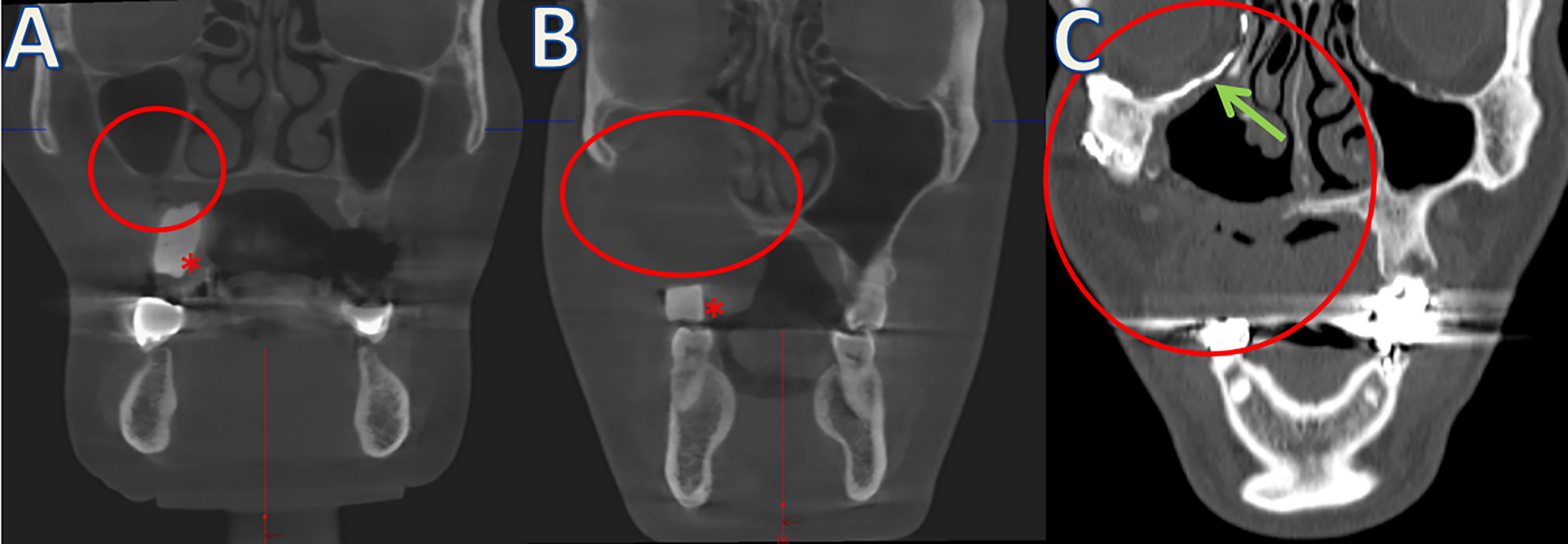
Examples of different Brown’s classifications (defect size marked with red circles). **(A)** Class 1 without oroantral fistula. **(B)** Class 2 after resection of parts of the maxillary sinus and covering with microvascular anastomosed soft tissue flaps. **(C)** Class 3 with resection of the orbital floor (arrow: patient-specific orbital implant). *Radiopaque scanning templates for prosthodontic backwards planning of IPS Implants^®^ Preprosthetic.

This is a retrospective single-center study, which included patients treated with a patient-specific implant (IPS Implants^®^ Preprosthetic) in course of secondary reconstruction after ablative surgery of the maxilla, at Hannover Medical School (Germany).The exclusion criteria were implant insertion without previous tumor resection, for example due to a failed augmentative procedure or a defect caused by trauma or cleft lip and palate. 20 of these patient-specific implants were included in this study, which were placed in 19 patients (10 males and 9 females) as one bilateral implantation was performed. The mean age of the patients at the time of surgery was 65 years (30–85 years). Most of the defects were due to a malignant tumor; in 65.75% of the cases, resection was performed due to squamous cell carcinoma, but rare tumor entities or benign lesions were also etiological for the defects. Depending on the underlying pathology, five patients received adjuvant radiotherapy prior to implant placement, and in most cases, primary reconstruction included free tissue transfer (**Table 1**). The defects were classified according to Brown’s classification, in which the number describes the vertical extent of the defect and the letter indicates the resected part of the alveolar process or palate, as shown in **Figure 1** (17).

**Table 1:** Synopsis of patients.

Diagnosis	Number of patients	Irradiation	Soft tissuefree flaps	Failed bone reconstruction	Time after primary surgery^1^
**Squamous cell carcinoma**	11	3	9	2	44.64 (37.10)
**Keratocyst**	2	–	1	1	20.50 (2.50)
**Mucoepidermoid carcinoma**	1	–	–	–	150
**Adenoid cystic carcinoma**	1	1	1	1	47
**Adenocarcinoma**	1	–	1	–	52
**Osteosarcoma**	1	–	1	1	91
**Malignant** **Melanoma**	1	1	1	–	9
**Myxoma**	1	–	1	–	39
**Total/Mean**	19	5	15	5	48.21 (39.72)

^1^in months; brackets. standard deviation.

**Figure 2:**
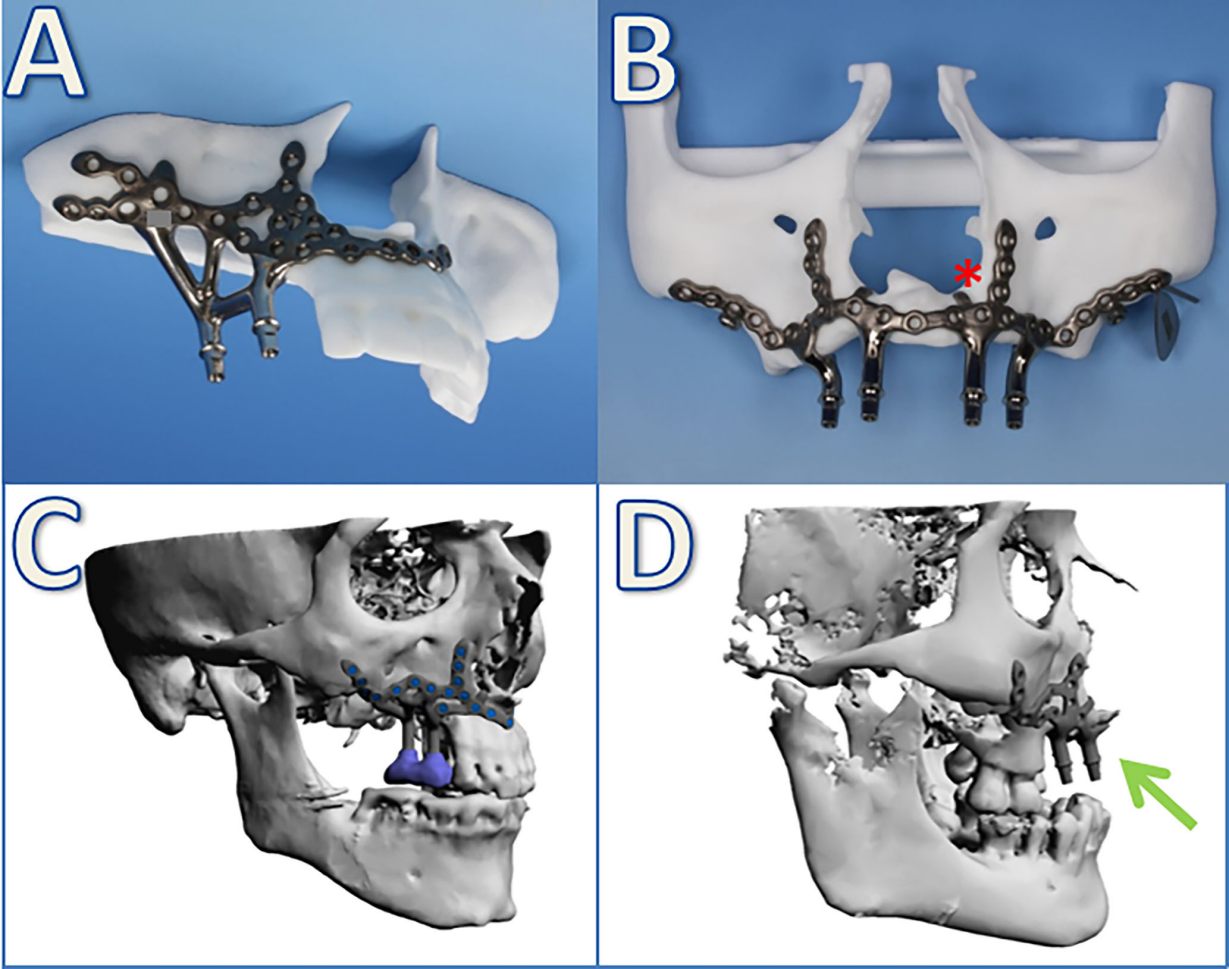
Examples of patient-specific implants from stereolithographic biomodels **(A, B)** as well as digital planning **(C, D)**. *Additional anatomical landmarks are included in the implant design as little flanges on both sides of the piriform aperture. Arrow: protrusion of the antagonizing implant against the massive (pseudo-) class III relationship.

The implants used for reconstruction were digitally backwards-planned based on the subsequent prosthodontic restoration as shown in **Figure 2**. The manufacturing process using selective laser melting allows 3D printing of a one-fit-only implant, which is placed subperiostally and anchored multi-vectorially by osteosynthesis screws, especially in the area of the load-bearing paranasal and lateral facial buttresses as well as implementing other features such as functionalization e.g. due to positioning aids in the area of the piriform aperture, tapering implant edges or a protruding pillar alignment to compensate for the maxillary tissue deficit. Depending on the defect or compromise of the load-bearing buttresses, the design of the patient-specific implant has to be adapted. With a higher Brown´s class 1 often a more distant anchorage is required, so that the implant is extended over the zygomatic arch, for example. The implementation of numerous screw holes guarantees multi-vector anchorage even in patients with poor bone quality. The authors prefer not to place under 20 screws, if this is possible.

Using a standardized protocol, the clinical implant stability as well as insertion of the definitive prosthodontic restoration were documented during follow-up as primary outcome variables. The duration of the operation, the number and size of the osteosynthesis screws, and the need for additional soft tissue procedures, such as local flaps, as well and complications were assessed as secondary variables. Descriptive statistical analyses were performed using Microsoft Excel 2010 and SigmaStat 4.0. Group comparisons were performed using the Fisher’s exact test. Statistical significance was set at *p* < 0.05, based on a 95% confidence interval. Written informed consent from the patients and ethical approval from the institutional ethics committee were obtained (reference number 8552_BO_K_2019).

## Results

Most of the patients were assigned to Brown’s class 2, suggesting that although the maxillary sinus was affected, the orbital floor remained intact. **Table 2** shows an overview of the extent of defects in the examined patient population.

**Table 2:** Defects according to Brown’s classification.

		a	b	c
**1**	3	–	–	–
**2**	–	6	6	1
**3**	–	–	3	–
**4**	–	–	–	–

**Figure 3:**
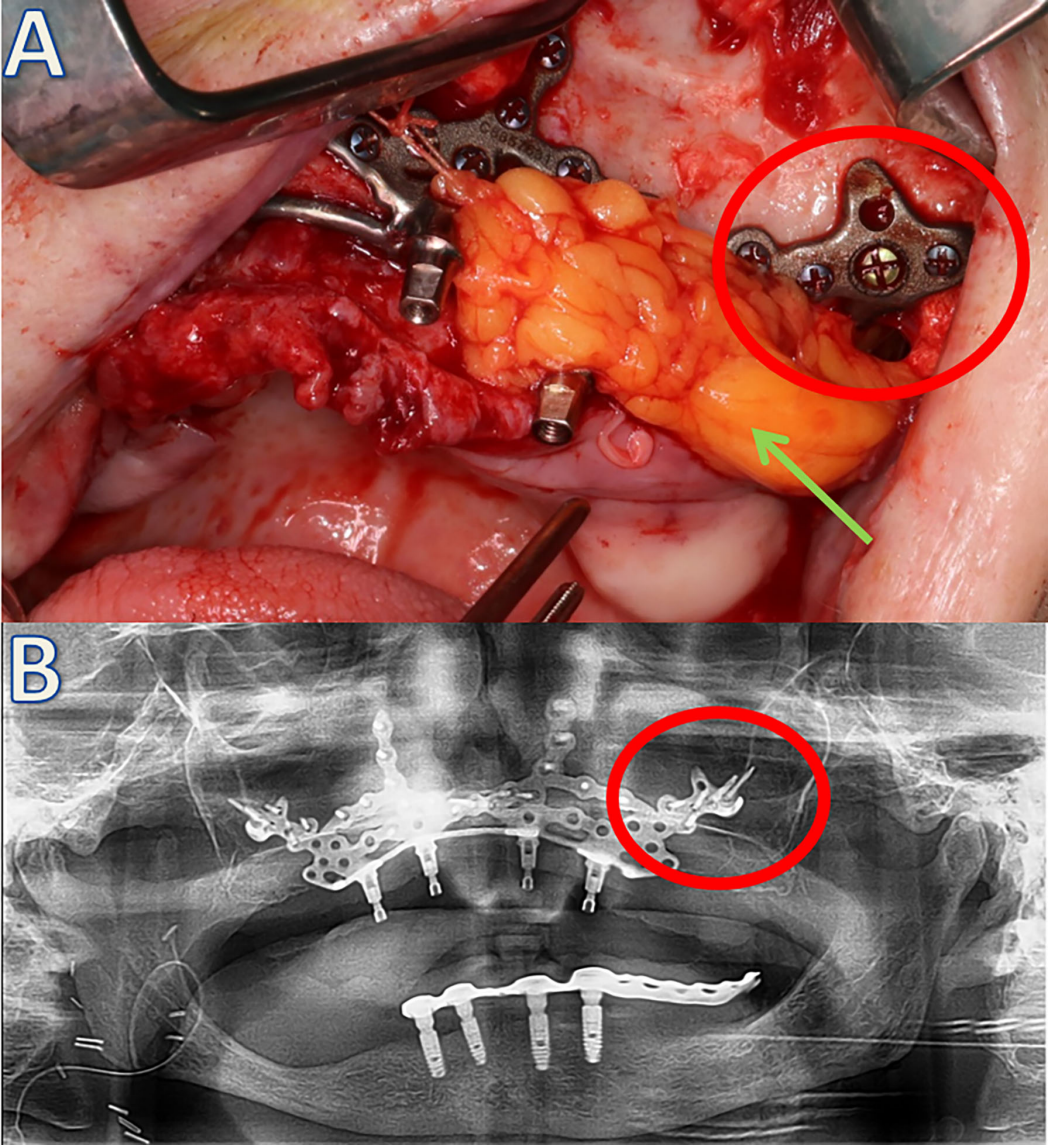
Soft tissue coverage of the left side using the Bichat’s fat pad (green arrow) **(A)** and postoperative orthopantomogram of the patient **(B)**. The red circle indicates the same implant area clinically and radiologically.

At the time of surgery, the average age of the patients was 65 years (30–87 years). The observation period was 6–74 months, with an average of 26 months. Adapted to the defect, the implants were designed with 2–4 posts. The average operating time was 127 min (69–205 min). A multivectorial screw-based anchoring of the subperiosteally placed implants was performed using an average of 22 (16–48) partly locking screws sized 1.2–2.0 mm, which always resulted in primary stability. In order to achieve soft tissue coverage of the implants, local flaps (n = 14), such as gingival advancement flaps (n = 11) were used just as regional flaps (n = 8), for example, by mobilization of Bichat’s fat pad (n = 6) or by using palatine artery flaps (n = 2) (**Figure 3**).

**Figure 4:**
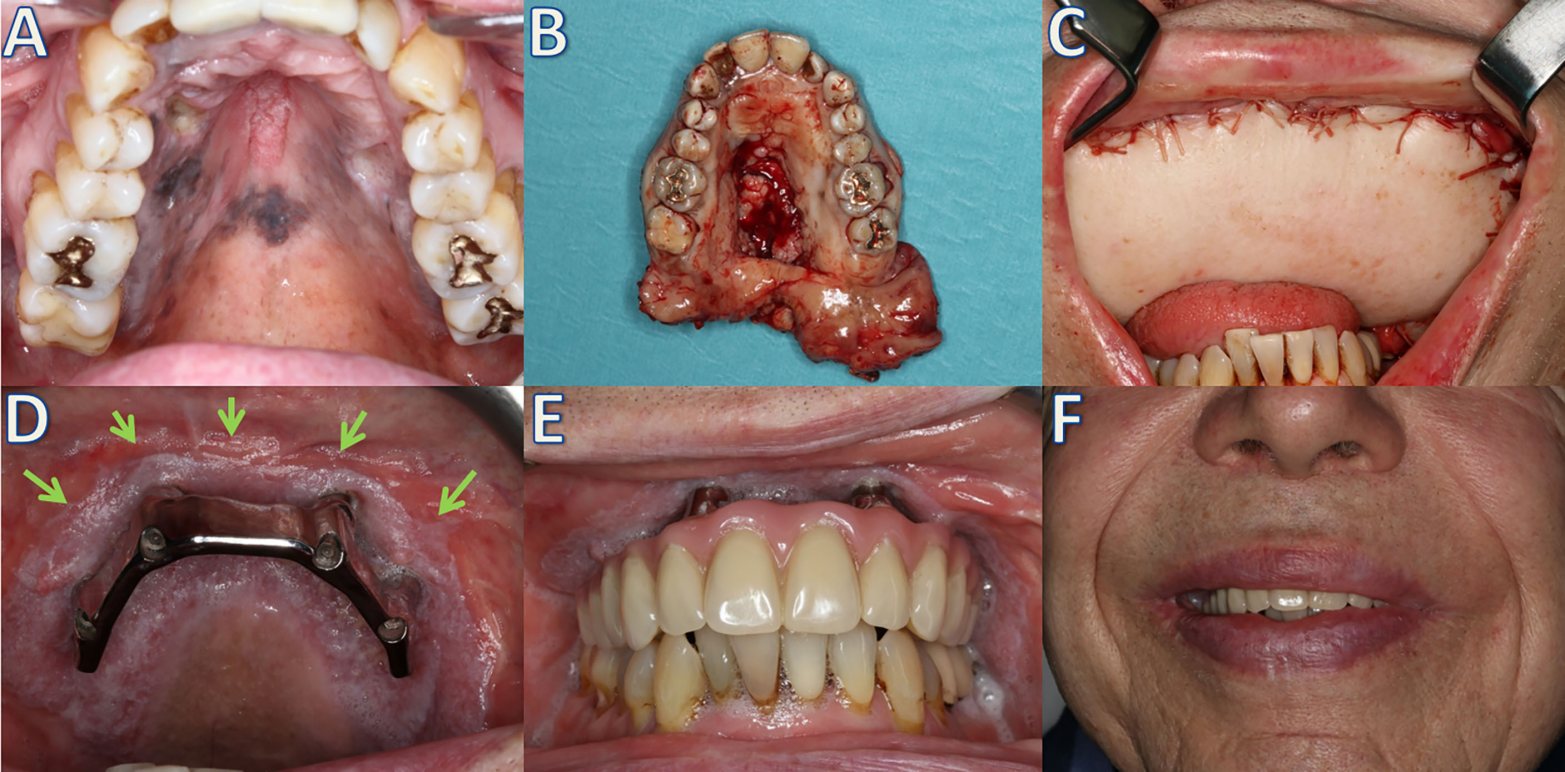
Patient with a multilocular malignant mucosal melanoma of the upper jaw **(A)**, which was treated with extended maxillectomy **(B)** and a latissimus dorsi free flap **(C)**. After irradiation, reconstruction using a patient-specific implant (IPS Implants^®^ Preprosthetic) and bar suprastructure; arrows: separation of anatomical units anterior to the implant posts **(D)**. Definitive palate-free prosthodontic restoration **(E)** and the clinical result **(F)**.

All implants showed clinical implant stability and were restored prosthetically. There were no clinical signs of implant loosening at any point of time, and no implant was lost. **Figure 4** shows a clinical case, from resection to the final prosthodontic restoration.

**Figure 5:**
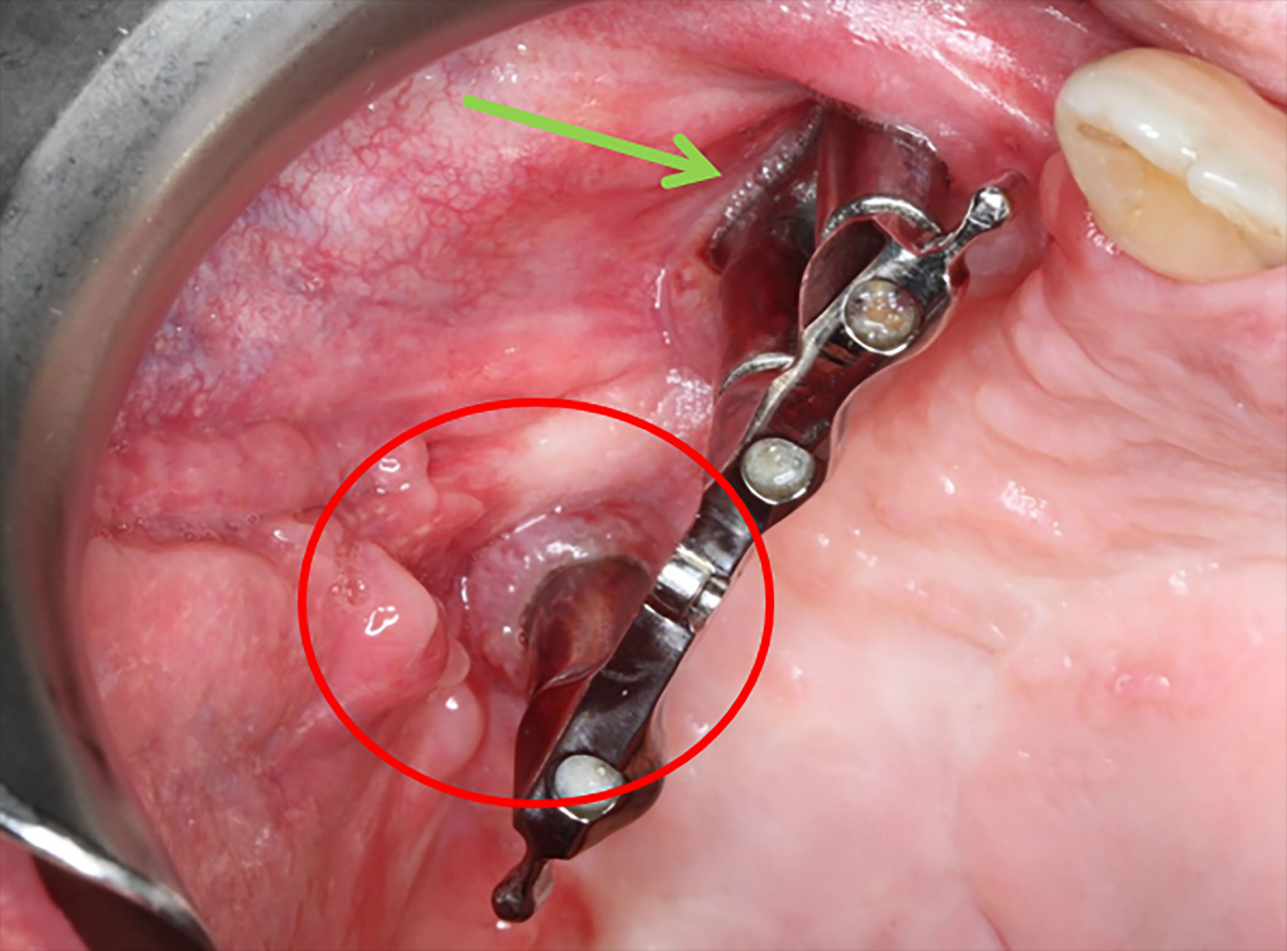
Minor complications during follow-up. IPS Implants^®^ preprosthetic with a bar-retained superstructure. The red circle shows mucositis of the soft tissue surrounding the dorsal post. The green arrow indicates an exposure of the underlying framework without any signs of inflammation.

In two cases, hardware related interventions had to be performed: in one case two exposed screws had to be removed as well as in the second case where in addition to screws a single implant post was reduced without affecting the stability of the implant or the prosthetic restoration per se. The implants are still in place, and patients are free of complaints. Partial exposure of the underlying framework was observed in nine cases (47.36%), without affecting implant stability or compromising a rapid prosthetic restoration (**Figure 5**). An additional soft tissue coverage using a radial free forearm flap was necessary in one case (with a history of radiotherapy in the course of tumor treatment). In the area of the soft tissue surrounding the implant posts that penetrate into the oral cavity, an inflammatory reaction in the form of mucositis was observed in some cases. The denture saddle should shield the soft tissues to prevent movement of the implant posts, especially if separation of the anatomical units is not guaranteed (**Figure 5**). Two postoperative infections in the form of abscesses were observed, both of which occurred in irradiated patients; one of the abscesses progressed to an extraoral fistula. A trend toward a higher rate of major complications in irradiated patients was observed (*p* = 0.058), without any influence on the endpoints, such as implant survival or final prosthetic restoration.

## Discussion

Autologous bone grafting and prosthodontic restoration using obturators represent the most commonly used techniques for post-ablative maxillary reconstruction (2). Both treatment options have individual disadvantages: in case of autologous bone grafts, there are issues of donor-site morbidity and risk of graft loss, and in case of obturators, nasal leakage as well discomfort (e.g., instability) may cause problems. Thus, valid recommendations regarding the best therapy, especially those concerning the quality of life, are still missing (18, 19). The use of patient-specific implants (IPS Implants^®^ Preprosthetic) offers a one-step palate-free implant-borne prosthodontic restoration without any donor-site morbidity. In addition, time-consuming and invasive augmentative procedures, which pose a risk of graft loss and are often refused by patients, are avoided. The only prerequisite for this approach to rehabilitate patients with extended maxillary defects is that the compromised anatomical units should be separated by soft tissue reconstruction prior to IPS preprosthetic implant placement. Depending on the individual defect size and extent of tissue damage, including post-radiation sequelae, the surgeon has to move up the soft tissue repair scale, from local flaps to microvascular soft tissue free flaps (fasciocutaneous or myocutaneous), in patients with extended maxillary defects. However, microvascular bone flaps donor sites when compared to microvascular soft tissue flaps are much fewer and donor-site morbidity is significantly higher (20, 21). In addition, after tumor resection, patients often shy away from complex reconstructions requiring tissue transplantation or augmentative procedures. Such patients require an alternative strategy similar to those with a history of failed reconstructions. Furthermore, many tumor patients are not eligible for major surgical interventions because of their reduced general condition. The excellent survival rates up to a follow-up period of up to 74 months indicate that this method is safe, with predictable results in terms of implant stability and definitive prosthodontic restoration. With regard to the quality of life, initial data show that restoration with patient-specific implants achieves outcomes that are comparable or even superior to those of conventional implants (22). This treatment option appears at first glance to be similar to the previously used subperiosteal implants, that were abandoned due to high complication rates (23). However, this is not true because the multivectorial and distant fixation of the implant using the midfacial buttresses or even the lateral skull base in selected cases fundamentally differs as an average of 22 screws achieves primary stability and allows full and immediate loading. Due to the possibilities of digital technologies, the abandoned idea of subperiosteally placed implants has been revisited and several approaches have been published (24–26), whereby individual designs and anchoring methods differ significantly. It is precisely this distant fixation as well as the multivectoral alignment of the comparatively large number of screws that distinguishes the implant system used from others that initially may appear similar. Particularly in compromised bone conditions, as in the patient group studied, anchoring with only a few screws appears dangerous, since patient-specific implants - unlike osteosynthesis plates, for example - cannot be repositioned due to their “one-fit-only” design if a screw fails to tighten.

Occasionally, signs of inflammation around the soft tissue surrounding the post were observed, but in contrast to conventional dental implants, where local infection may lead to implant loss *via* peri-implantitis, this does not seem to occur in IPS preprosthetic implants. The posts are not anchored directly in the bone and due to the distant fixation by screws, inflammation seems to remain limited to the soft tissue only. In contrast to osseointegrated conventional implants mucositis does not spread to the bone. This may be one reason that long-term stable conditions could be achieved even with recurrent episodes of mucosal inflammation and superficial exposure of framework structures. Severe postoperative complications, such as abscesses or dehiscence, requiring additional microvascular tissue transfer, only occurred in previously irradiated patients in whom the need for soft tissue was primarily underestimated. The principle of separating the anatomical units prior to IPS implant placement has to be strictly followed. As well as in alternative procedures, a history of irradiation leads to a higher rate of complications (27, 28).

Zygomatic implants used for implant-borne prosthodontic restoration are quite technology-dependent and often require an additional equipment, such as intraoperative real-time navigation (29). Furthermore, multiple complications such as sinusitis or incorrect positioning have been described (30, 31). This did not occur in the present study. Since the IPS preprosthetic implants are inserted under direct vision, there is no need for such technical prerequisites. The technical requirements for planning patient-specific implants are also shifted from the surgeon toward industrial partners. Furthermore, 3D features such as flanges toward the piriform aperture or around the transition zone between the malar prominence and zygomatic arch guarantee a one-fit-only position of IPS preprosthetic implants, in contrast to conventional dental or zygomatic implants. In addition, the possibility of positioning the implant shoulder of zygomatic implants is only possible up to the molar or premolar region and not in the esthetic zone. Zygomatic implants also do not offer a real solution to compensate for the severe postablative (pseudo-) skeletal class III relation. Owing to the patient-specific design of the IPS preprosthetic implant, a massive protrusion of the implant posts is feasible, especially in the anterior part of the (former) maxilla, enabling a functionally and esthetically satisfactory result. In contrast to restoration with conventional dental implants, the posts are aligned parallel from planning to implant insertion and final restoration. The noticeably shorter and less invasive surgical procedure compared to microvascular tissue transfer were noted, with an average duration of approximately 2 hours. Thus treatment as an outpatient is feasible.

Postablative maxillary defects often result in facial disfigurement and compromised function (6). Patients’ desire for rapid functional and esthetic rehabilitation is often not possible with microvascular bone transplantation. After tumor resection or adjuvant therapy (in some cases), patients are often tired of treatment and shy away from complex reconstructions, especially after failure of tissue transfer or augmentation. Nevertheless, there is an understandable desire for dental rehabilitation with fixed palate-free dentures. The treatment algorithm presented here provides a predictable reconstruction, even after extended maxillary ablation, without time-consuming bone augmentation. With irradiated patients, the complication rate appears to increase, although all patients examined in this study were successfully rehabilitated. The patient cohort in this study is small, although the results are encouraging and the portfolio for the treatment of postablative maxillary defects is expanded. Especially if microvascular bone grafting seems not feasible or patients refuse such a procedure, secondary reconstruction using a patient-specific implant may be an alternative option.

## Data Availability Statement

The original contributions presented in the study are included in the article/supplementary material. Further inquiries can be directed to the corresponding author.

## Ethics Statement

The studies involving human participants were reviewed and approved by Ethikkommission der Medizinischen Hochschule Hannover Hannover Medical School, Hannover, Germany. The patients/participants provided their written informed consent to participate in this study. Written informed consent was obtained from the individual(s) for the publication of any potentially identifiable images or data included in this article.

## Author Contributions

PK and N-CG designed the study and drafted the manuscript. BR analysed the data and revised the manuscript. PJ and SS revised the article for important intellectual content. All authors contributed to the article and approved the submitted version.

## Conflict of Interest

N-CG and BR received honoraria for speaking or traveling from KLS Martin.

The remaining authors declare that the research was conducted in the absence of any commercial or financial relationships that could be construed as a potential conflict of interest.

## Publisher’s Note

All claims expressed in this article are solely those of the authors and do not necessarily represent those of their affiliated organizations, or those of the publisher, the editors and the reviewers. Any product that may be evaluated in this article, or claim that may be made by its manufacturer, is not guaranteed or endorsed by the publisher.

## References

[1] Brown JS Shaw RJ. Reconstruction of the Maxilla and Midface: Introducing a New Classification. Lancet Oncol (2010) 11:1001–8. doi: 10.1016/S1470-2045(10)70113-3 20932492

[2] Andrades P Militsakh O Hanasono MM Rieger J Rosenthal EL. Current Strategies in Reconstruction of Maxillectomy Defects. Arch Otolaryngol Head Neck Surg (2011) 137:806–12. doi: 10.1001/archoto.2011.132 PMC3951334 21844415

[3] Jung S Kook M-S Park H-J Oh H-K. Delayed Closure of the Palatal Defect Using Buccal Inversion and Palatal Rotation Flaps After Maxillectomy. J Craniofac Surg (2013) 24:660–4. doi: 10.1097/SCS.0b013e31827c8611 23524771

[4] Vincent A Burkes J Williams F Ducic Y. Free Flap Reconstruction of the Maxilla. Semin Plast Surg (2019) 33:30–7. doi: 10.1055/s-0039-1677701 PMC6408236 30863210

[5] Parr GR Gardner LK. The Evolution of the Obturator Framework Design. J Prosthet Dent (2003) 89:608–10. doi: 10.1016/s0022-3913(03)00176-8 12815356

[6] Lenox ND Kim DD. Maxillary Reconstruction. Oral Maxillofac Surg Clin North Am (2013) 25:215–22. doi: 10.1016/j.coms.2013.01.004 23642669

[7] Dahl G. Om MöJligheten FöR Inplantation I KäKen Avmetallskelett Som Bas Eller Retention FöR Fasta Eller Avtagbaraproteser. Odontologisk Tidskrift (1943) 51:440–9.

[8] Goldberg NI Gershkoff A. The Implant Lower Denture. Dental Digest (1949) 55:490–5. 15395224

[9] Rohner D Kunz C Bucher P Hammer B Prein J. New Possibilities for Reconstructing Extensive Jaw Defects With Prefabricated Microvascular Fibula Transplants and ITI Implants. Mund Kiefer Gesichtschir (2000) 3:365–72. doi: 10.1007/s100060000228 11151343

[10] Rohner D Jaquiéry C Kunz C Bucher P Maas H Hammer B. Maxillofacial Reconstruction With Prefabricated Osseous Free Flaps: A 3-Year Experience With 24 Patients. Plast Reconstr Surg (2003) 112:748–57. doi: 10.1097/01.PRS.0000069709.89719.79 12960855

[11] Rana M Chin S-J Muecke T Kesting M Groebe A Riecke B. Increasing the Accuracy of Mandibular Reconstruction With Free Fibula Flaps Using Functionalized Selective Laser-Melted Patient-Specific Implants: A Retrospective Multicenter Analysis. J Craniomaxillofac Surg (2017) 45:1212–9. doi: 10.1016/j.jcms.2017.04.003 28552201

[12] Weitz J Wolff K-D Kesting MR Nobis C-P. Development of a Novel Resection and Cutting Guide for Mandibular Reconstruction Using Free Fibula Flap. J Craniomaxillofac Surg (2018) 46:1975–8. doi: 10.1016/j.jcms.2018.09.007 30293853

[13] Zavattero E Bolzoni A Dell’Aversana G Santagata M Massarelli O Ferri A. Accuracy of Fibula Reconstruction Using Patient-Specific Cad/Cam Plates: A Multicenter Study on 47 Patients. Laryngoscope (2021) 131:E2169–75. doi: 10.1002/lary.29379 33452834

[14] Hackett S El-Wazani B Butterworth C. Zygomatic Implant-Based Rehabilitation for Patients With Maxillary and Mid-Facial Oncology Defects: A Review. Oral Dis (2021) 27:27–41. doi: 10.1111/odi.13305 32048429

[15] Gellrich N-C Zimmerer RM Spalthoff S Jehn P Pott P-C Rana M. A Customised Digitally Engineered Solution for Fixed Dental Rehabilitation in Severe Bone Deficiency: A New Innovative Line Extension in Implant Dentistry. J Craniomaxillofac Surg (2017) 45:1632–8. doi: 10.1016/j.jcms.2017.07.022 28867525

[16] Gellrich N-C Rahlf B Zimmerer R Pott P-C Rana M. A New Concept for Implant-Borne Dental Rehabilitation; How to Overcome the Biological Weak-Spot of Conventional Dental Implants? Head Face Med (2017) 13:17. doi: 10.1186/s13005-017-0151-3 28962664 PMC5622522

[17] Brown JS Rogers SN McNally DN Boyle M. A Modified Classification for the Maxillectomy Defect. Head Neck (2000) 22:17–26. doi: 10.1002/(sici)1097-0347(200001)22:1<17::aid-hed4>3.0.co;2-2 10585601

[18] Cao Y Yu C Liu W Miao C Han B Yang J. Obturators Versus Flaps After Maxillary Oncological Ablation: A Systematic Review and Best Evidence Synthesis. Oral Oncol (2018) 82:152–61. doi: 10.1016/j.oraloncology.2018.05.019 29909890

[19] Buurman DJM Speksnijder CM de Groot RJ Kessler P Rieger JM. Mastication in Maxillectomy Patients: A Comparison Between Reconstructed Maxillae and Implant Supported Obturators: A Cross-Sectional Study. J Oral Rehabil (2020) 47:1171–7. doi: 10.1111/joor.13043 PMC7497273 32613633

[20] Ling XF Peng X Samman N. Donor-Site Morbidity of Free Fibula and DCIA Flaps. J Oral Maxillofac Surg (2013) 71:1604–12. doi: 10.1016/j.joms.2013.03.006 23810616

[21] Kansy K Hoffmann J Alhalabi O Mistele N Freier K Shavlokhova V. Long-Term Donor Site Morbidity in Head and Neck Cancer Patients and its Impact on Quality of Life: A Cross-Sectional Study. Int J Oral Maxillofac Surg (2019) 48:875–85. doi: 10.1016/j.ijom.2019.01.009 30718032

[22] Jehn P Spalthoff S Korn P Stoetzer M Gercken M Gellrich N-C. Oral Health-Related Quality of Life in Tumour Patients Treated With Patient-Specific Dental Implants. Int J Oral Maxillofac Surg (2020) 49:1067–72. doi: 10.1016/j.ijom.2020.01.011 31992467

[23] Schou S Pallesen L Hjørting-Hansen E Pedersen CS Fibaek B. A 41-Year History of a Mandibular Subperiosteal Implant. Clin Oral Implants Res (2000) 11:171–8. doi: 10.1034/j.1600-0501.2000.110210.x 11168208

[24] Van den Borre C Rinaldi M De Neef B Loomans NAJ Nout E Van Doorne L. Radiographic Evaluation of Bone Remodeling After Additively Manufactured Subperiosteal Jaw Implantation (AMSJI) in the Maxilla: A One-Year Follow-Up Study. J Clin Med (2021) 10:3542. doi: 10.3390/jcm10163542 34441837 PMC8397126

[25] Cerea M Dolcini GA. Custom-Made Direct Metal Laser Sintering Titanium Subperiosteal Implants: A Retrospective Clinical Study on 70 Patients. BioMed Res Int (2018) 2018:5420391. doi: 10.1155/2018/5420391 29998133 PMC5994585

[26] Mangano C Bianchi A Mangano FG Dana J Colombo M Solop I. Custom-Made 3D Printed Subperiosteal Titanium Implants for the Prosthetic Restoration of the Atrophic Posterior Mandible of Elderly Patients: A Case Series. 3D Print Med (2020) 6:1. doi: 10.1186/s41205-019-0055-x 31915946 PMC6950914

[27] Schmidt BL Pogrel MA Young CW Sharma A. Reconstruction of Extensive Maxillary Defects Using Zygomaticus Implants. J Oral Maxillofac Surg (2004) 62(9 Suppl 2):82–9. doi: 10.1016/j.joms.2004.06.027 15332185

[28] Lodders JN Leusink FKJ Ridwan-Pramana A Winters HAH Karagozoglu KH Dekker H. Long-Term Outcomes of Implant-Based Dental Rehabilitation in Head and Neck Cancer Patients After Reconstruction With the Free Vascularized Fibula Flap. J Craniomaxillofac Surg (2021) 30:S1010–5182(21)00101-3. doi: 10.1016/j.jcms.2021.03.002 33985871

[29] Schramm A Gellrich NC Schimming R Schmelzeisen R. [Computer-Assisted Insertion of Zygomatic Implants (Brånemark System) After Extensive Tumor Surgery]. Mund Kiefer Gesichtschir (2000) 4:292–5. doi: 10.1007/s100060000211 11092181

[30] Molinero-Mourelle P Baca-Gonzalez L Gao B Saez-Alcaide L-M Helm A Lopez-Quiles J. Surgical Complications in Zygomatic Implants: A Systematic Review. Med Oral Patol Oral Cir Bucal (2016) 21:e751–7. doi: 10.4317/medoral.21357 PMC5116118 27694789

[31] D’Agostino A Lombardo G Favero V Signoriello A Bressan A Lonardi F. Complications Related to Zygomatic Implants Placement: A Retrospective Evaluation With 5 Years Follow-Up. J Craniomaxillofac Surg (2021) 5:S1010–5182(21)00033-0. doi: 10.1016/j.jcms.2021.01.020 33581959

